# Safety and immunogenicity of a delta inulin-adjuvanted inactivated Japanese encephalitis virus vaccine in pregnant mares and foals

**DOI:** 10.1186/s13567-014-0130-7

**Published:** 2014-12-17

**Authors:** Helle Bielefeldt-Ohmann, Natalie A Prow, Wenqi Wang, Cindy SE Tan, Mitchell Coyle, Alysha Douma, Jody Hobson-Peters, Lisa Kidd, Roy A Hall, Nikolai Petrovsky

**Affiliations:** School of Veterinary Science, University of Queensland, Gatton Campus, Gatton, Qld 4343 Australia; Australian Infectious Diseases Research Centre, University of Queensland, St. Lucia, Qld 4078 Australia; School of Chemistry & Molecular Biosciences, University of Queensland, St. Lucia, Australia; Gatton Campus Equine Unit, University of Queensland, Gatton Campus, Gatton, Qld 4343 Australia; Vaxine Pty., Ltd., Flinders Medical Centre, Adelaide, South Australia; Flinders Medical Centre and Flinders University, Bedford Park, South Australia

## Abstract

In 2011, following severe flooding in Eastern Australia, an unprecedented epidemic of equine encephalitis occurred in South-Eastern Australia, caused by Murray Valley encephalitis virus (MVEV) and a new variant strain of Kunjin virus, a subtype of West Nile virus (WNV_KUN_). This prompted us to assess whether a delta inulin-adjuvanted, inactivated cell culture-derived Japanese encephalitis virus (JEV) vaccine (JE-ADVAX™) could be used in horses, including pregnant mares and foals, to not only induce immunity to JEV, but also elicit cross-protective antibodies against MVEV and WNV_KUN_. Foals, 74–152 days old, received two injections of JE-ADVAX™. The vaccine was safe and well-tolerated and induced a strong JEV-neutralizing antibody response in all foals. MVEV and WNV_KUN_ antibody cross-reactivity was seen in 33% and 42% of the immunized foals, respectively. JE-ADVAX™ was also safe and well-tolerated in pregnant mares and induced high JEV-neutralizing titers. The neutralizing activity was passively transferred to their foals via colostrum. Foals that acquired passive immunity to JEV via maternal antibodies then were immunized with JE-ADVAX™ at 36–83 days of age, showed evidence of maternal antibody interference with low peak antibody titers post-immunization when compared to immunized foals of JEV-naïve dams. Nevertheless, when given a single JE-ADVAX™ booster immunization as yearlings, these animals developed a rapid and robust JEV-neutralizing antibody response, indicating that they were successfully primed to JEV when immunized as foals, despite the presence of maternal antibodies. Overall, JE-ADVAX™ appears safe and well-tolerated in pregnant mares and young foals and induces protective levels of JEV neutralizing antibodies with partial cross-neutralization of MVEV and WNV_KUN_.

## Introduction

Flaviviruses of the Japanese encephalitis virus (JEV) sero-complex are amongst the most important encephalitic viruses worldwide, affecting humans, wild birds, and several mammalian species, including domestic animal species such as horses. JEV is the leading cause of viral encephalitis in Asia, where 2–3 billion people are at risk of contracting the disease [1,2]. Annually, ~35 000 cases of JE are reported with a case fatality rate of nearly 30% and more than 50% of the survivors having neurological sequelae. Clinical manifestations vary and may include fever, headache, a change in mental status, seizures, tremors, generalised paresis, hypertonia and loss of coordination [3]. The clinical course in horses resembles that found in humans [4-7] with the majority of equine JEV infections being subclinical [8]. There is an estimated incidence of JE of 0.05% of JEV infections with a JE case fatality rate of ~50%. Treatment of JE patients, whether humans or horses, in the absence of availability of antiviral compounds, is supportive and the best means of preventing JE is immunization [9].

An inactivated JEV vaccine, developed in Japan in the 1960s (JE-VAX), dramatically reduced the number of human and equine cases of JE in that country [10]. However, this vaccine ceased to be manufactured in 2005, due to perceived safety problems and excessive reactogenicity, with subsequent JEV vaccines being developed based on inactivated virus grown in cell culture [10,11].

Vaccination of thoroughbred horses against JEV is mandatory in several Asian countries. However, in many countries there is currently no widely available and approved equine JEV vaccine resulting in potential off-label human vaccine use in horses. For example, vaccine failure and fatal encephalitis due to naturally acquired JEV infection has been reported in a racing horse imported from Australia into Hong Kong [5]. Cases of equine JE have the potential to cause significant adverse economic effect on the horse industry, which is estimated to contribute greater than $6 billion to the GDP in Australia alone [12].

An inactivated Vero cell culture-derived JEV vaccine combined with delta inulin adjuvant (JE-ADVAX™) was previously tested in mice and adult horses and shown to have superior immunogenicity compared to the now-discontinued JE-VAX as well as a recently licensed, alum-adjuvanted cell culture-derived vaccine (JESPECT®, Novartis) [13]. The primary aim of the present study was to undertake vaccine efficacy and safety trials of the new JE-ADVAX™ vaccine in pregnant mares and in foals without or with passively acquired maternal antibodies. A second aim was to explore the potential ability of the JE-ADVAX™ vaccine to induce cross-reactivity and cross-protection against two related viruses, Murray Valley encephalitis virus (MVEV) and a new equine-virulent WNV_KUN_ strain (strain NSW2011), which appeared in South-East Australia in early 2011 and caused a large epidemic of equine encephalitis [14,15]. Given the unlikely future development of equine vaccines specifically against MVEV and WNV_KUN_, it would be useful if an adjuvanted JEV vaccine could provide cross-protection against these related flaviviruses.

In the present report we demonstrate safety and efficacy of the JE-ADVAX™ vaccine in young foals and pregnant mares. Foals born to unvaccinated mares responded to the vaccine with long-lasting humoral immunity, while foals with passively acquired maternal JEV antibodies had a blunted response to primary immunization, but after a vaccine booster as yearlings had a robust JEV-specific response indicating that memory B cells had been successfully primed during the primary immunization despite interference from maternal antibodies.

## Materials and methods

### Antigen and adjuvant

The Vero cell culture-grown inactivated JEV vaccine (Beijing-1 strain) [16] was obtained from the Kitasato Institute, Japan. The Advax™ adjuvant was described in detail in Lobigs et al. [13]. Briefly, Advax adjuvant is based on microparticulate delta inulin [17], and was obtained from Vaxine Pty Ltd, Adelaide, Australia. Advax™ is supplied as a sterile, preservative-free, fine particulate suspension of delta inulin particles in a phosphate buffer. The vaccine antigen and Advax adjuvant were mixed together less than two hours prior to inoculation and the mixture kept on wet ice until injected.

### Animals, vaccination protocol and sample collection

The studies were approved by the University of Queensland Animal Ethics Committee (AEC nos. SVS/306/11/VAXINE and SVS/298/13/VAXINE), and carried out in accordance with the ARC/NHMRC guidelines for ethical use of animals in research. A total of 53 thoroughbred and standard bred horses were enrolled in the studies (Tables 1, 2, 3 and 4). All horses were held in paddocks at the UQ Gatton Campus throughout the studies and received supplementary feed when needed. The first cohort of 19 foals, 74–152 days of age at first vaccination, were born to non-vaccinated mares and were all sero-negative for flavivirus antibodies at entry into the trial (Table 3). Twelve foals were vaccinated with the Advax-adjuvanted JEV-vaccine, while seven foals received the adjuvant only. Seventeen mares in the second trimester of pregnancy were enrolled in the second phase, with 11 mares receiving the JEV-antigen plus Advax and six mares receiving adjuvant only. Two of the JEV-vaccinated mares had pre-existing antibodies specific for flaviviruses other than JEV, MVEV or WNV (data not shown; [18]). The foals born to the mares were subsequently enrolled in the third phase of the study (see below).

**Table 1:** **Flavivirus specific antibody levels in foals born to flavivirus antibody-negative mares and vaccinated at 74–152 days of age** ^**a**^

	**Age at**	**JEV neutralising antibodies**			**MVEV neutralising antibodies**			**WNV**_**KUN**_ **neutralising antibodies**		
**Foal #**	**Day 0**	**Day 28 (boost)**	**Day 56**	**Day 308**	**Day 28 (boost)**	**Day 56**	**Day 308**	**Day 28 (boost)**	**Day 56**	**Day 308**
1 F11	74		**80**	**20**		<20	< 20		< 20	**20**
5 F11	115	**40**	**160**	**160**	< 20	<20	**20**	< 20	< 20	< 20
7 F11	108		**160**	**40**		<20	< 20		**40**	< 20
8 F11	132		**160**	< 20		**20**	< 20		**40**	< 20
11 F11	119		**160**	**80**		**20**	< 20		**40**	< 20
12 F11	81	**40**	**1280**	**80**	< 20	**80**	< 20	< 20	**80**	< 20
14 F11	123		**320**	**20**		<20	**20**		**20**	< 20
15 F11	125		**320**	< 20		<20	< 20		**40**	**20**
17 F11	111		**320**	**20**		<20	< 20		< 20	< 20
21 F11	97		**320**	< 20		**20**	< 20		< 20	**20**
26 F11	116	**40**	**1280**	**80**	20	**80**	**20**	< 20	< 20	**20**
30 F11	111		**160**	**20**		<20	< 20		< 20	**20**
Control A	115		< 20	< 20		<20	**20**		< 20	**20**
Control G	115		< 20	< 20		<20	< 20		< 20	< 20

^a^Samples negative in the flavivirus (4G2) blocking ELISA were not tested in the neutralization assays. This included five control foals (B-F), aged 125 to 152 days, which are not listed in the table.

**Table 2:** **Flavivirus antibody responses in mares vaccinated in second trimester of pregnancy**

**Time point**	**JEV neutralizing**			**MVEV neutralizing**			**WNV**_**KUN**_ **neutralizing**		
	**Positive/total**	**Mean titre** ^**≠**^	**Positive titre range**	**Positive/total**	**Mean titre** ^**≠**^	**Positive titre range**	**Positive/total**	**Mean titre** ^**≠**^	**Positive titre range**
**Day 0 (vaccination)**	0/11	--	--	0/11	--	--	0/11	--	--
**Day 28 (booster)**	0/11	--	--	0/11	--	--	2/11	80	20-80
**Days 56-183**	8/11	180	20-320	4/11	100	20-160	3/11	560	20-1280
**Day 230 (7.5 months)**	11/11	47	20-80	9/11	31	20-80	2/11*	60	20-80
**Day 331 (11 months)**	7/11	26	20-40	4/11	40	20-80	2/11*	60	20-80

^**≠**^mean of titres ≥ 20.

*same two mares (# 10 & 33).

**Table 3:** **Flavivirus-specific antibodies in colostrum and serum of JE + ADVAX™** **vaccinated and unvaccinated mares at the time of foaling** ^**a**^

	**Colostrum**			**Serum**		
**Mare #**	**JEV neutralizing antibody titres**	**MVEV neutralizing antibody titres**	**WNV**_**KUN**_ **neutralizing antibody titres**	**JEV neutralizing antibody titres**	**MVEV neutralizing antibody titres**	**WNV**_**KUN**_ **neutralizing antibody titres**
2	2560	80	< 40	40	< 20	< 20
5	160	< 40	< 40	20	20	< 20
8	640	80	< 40	20	< 20	< 20
10	640	80	80	40	20	80
11	320	40	< 40	< 20	20	< 20
14	640	80	< 40	20	< 20	< 20
17	320	40	< 40	40	40	40
21	160	40	< 40	< 20	< 20	< 20
26	160	80	< 40	20	< 20	< 20
33	640	80	40	40	40	80
34	640	< 40	< 40	40	< 20	< 20
Control A^b^	160	40	< 40	< 20	< 20	< 20
Control B	40	40	< 40	< 20	< 20	< 20
Control C	320	40	< 40	< 20	< 20	< 20

^a^Samples negative in the flavivirus (4G2) blocking ELISA were not tested in the neutralization assays. This included three control mares (D-F), which are not shown in the Table.

^b^Entered trial at time of foaling, having received neither JEV + Advax nor Advax alone.

**Table 4:** **Flavivirus specific antibody levels in foals born to vaccinated mares and vaccinated at 36–83 days of age, followed by booster vaccinations four weeks and approximately one year later**

**Time point**	**JEV neutralizing**			**MVEV neutralizing**			**WNV**_**KUN**_ **neutralizing**		
	**Positive/total**	**Mean titre** ^**≠**^	**Titre range**	**Positive/total**	**Mean titre** ^**≠**^	**Titre range**	**Positive/total**	**Mean titre** ^**≠**^	**Titre range**
**Pre-suckle**	0/11	--	--	0/11	--	--	0/11	--	--
**Post suckle**	10/11	46	< 20-80	8/11	30	< 20-40	3/11	93	< 20-160
**Age 13–49 days**	3/10*	26	< 20-40	1/10*	40	< 20-40	2/10*	30	< 20-40
**Vaccination**	3/11	20	< 20-20	2/11	20	< 20-20	1/11	40	< 20-40
**Age 36–83 days**									
**Booster 4 weeks post vaccination**	2/11	20	< 20-20	3/11	20	< 20-20	2/11	20	< 20-20
**Age 64–111 days**									
**4 weeks post booster**	10/11	88	< 20-320	10/11	30	< 20-40	1/11	20	< 20-20
**Age 93–140 days**									
**7 weeks post booster**	3/11	53	< 20-80	6/11	26	< 20-40	3/11	20	< 20-20
**Age 114–161 days**									
**10 weeks post booster**	5/11	24	< 20-40	0/11	-	< 20	1/11	20	< 20-20
**Age 137–184 days**									
**~9 months post 1** ^**st**^ **boost (2** ^**nd**^ **boost)**	0/11	--	< 20	0/11	--	< 20	0/11	--	< 20
**Age 332–379 days**									
**2 weeks post 2** ^**nd**^ **boost**	11/11	> 902	160- > 2560	10/11	50	< 20-80	1/11	20	< 20-20
**Age 346–393 days**									
**5.5 weeks post 2** ^**nd**^ **boost**	11/11	465	160-1280	7/11	17	< 20-40	0/11	-	< 20
**Age 370–417 days**									
**14 weeks post 2** ^**nd**^ **boost**	8/11	150	< 20-640	0/11	-	< 20	9/11	31	< 20-80
**Age 454-501**									
**20 weeks post 2** ^**nd**^ **boost**	7/11	63	< 20-160	0/11	-	< 20	0/11	-	< 20
**Age 498–545 days**									

*one foal not yet born at this sampling point.

^**≠**^mean of titres ≥ 20.

**Figure 1:**
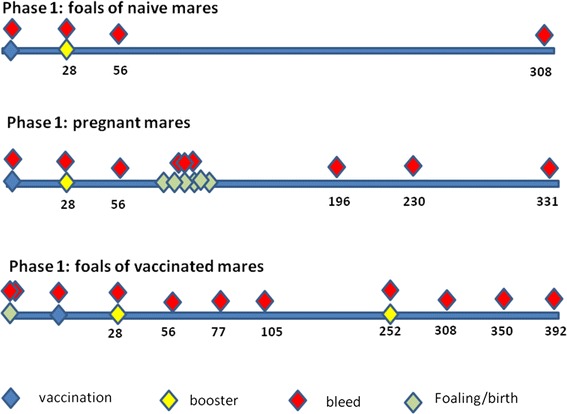
**Schematic of the vaccination schedules for the three cohorts.** The numbers under each line refer to days post-vaccination. The timelines are not to scale.

Vaccination was by subcutaneous inoculation on the rump (first foal cohort) or neck (mares and second foal cohort) in a volume of 150 μL. For both foals and mares the initial vaccine dose was 12 μg JEV-antigen plus 20 mg Advax™ and the booster vaccination, given four weeks later was 6 μg JEV-antigen and 20 mg Advax™. Blood samples were collected at each vaccination event and then at intervals of 4–36 weeks for up to 10 months post vaccination (Figure 1). The foals born to vaccinated mares were bled at birth, before colostrum uptake, and again 12 h after colostrum uptake, and a colostrum sample was obtained from the mares at foaling. This second cohort of foals was initially vaccinated at 36–83 days of age with a schedule similar to the first foal-cohort. They subsequently also received one additional vaccine booster (6 μg JEV-antigen and 20 mg Advax™) 10 months after the initial vaccination and blood samples were collected 2, 6, 12 and 18 weeks post vaccination. The six foals born to Advax™-only treated control mares were left untreated. Two of these unvaccinated foals were subsequently lost due to study-unrelated causes (birth complications and trauma, respectively).

Following each vaccine injection the animals were clinically assessed daily for 3–4 days while kept in small holding paddocks. The injection sites were inspected and any local reaction recorded. Body temperature and general demeanor were also recorded. With no adverse reactions recorded the animals were then returned to the main paddocks.

### Flavivirus serology

Blood samples were collected by venepuncture into sterile vacutainers (BD Biosciences, Franklin Lakes, NJ, USA) and allowed to clot at room temperature, then centrifuged at 4 °C for 10 min at 3500 rpm. Procured sera were stored at −20 °C in sterile cryovials until assayed by ELISA and virus neutralization following heat inactivation at 56 °C for 30 min. Colostrum samples were centrifuged and the non-fat fraction collected and heat-treated as for serum before testing in the ELISA and neutralization assay.

All horse sera and colostrum samples were initially screened for flavivirus-specific antibodies using an epitope-blocking ELISA [19,20] with minor modifications as described in detail in Prow et al. [18]. Samples showing > 30% inhibition in this assay were subsequently tested for neutralizing antibody reactivity to JEV (strain Nakayama), MVEV (strain 1–51) and WNV_KUN_ (strain NSW2011). The heat-inactivated test sera were titrated in doubling dilutions from 1:20 to 1:2560 and colostrum samples in dilutions from 1:40 to 1:5120 as previously described in detail [18].

## Results

### JE-ADVAX™ responses in foals born to naïve mares

In this trial, 19 foals, aged 74–152 days and born to non-vaccinated, flavivirus sero-negative mares, were enrolled. Twelve foals received two vaccinations four weeks apart with 12 μg and 6 μg JE-ADVAX™, respectively. Six control foals received ADVAX™ alone without antigen. Only mild swelling at the inoculation site was noted in some of the foals 1–3 days following subcutaneous injection on the rump. No reaction was seen in the Advax-only treated foals. Three foals had flavivirus-specific antibodies at the time of vaccine-boosting (Table 1), though none had virus-neutralizing activity. However, four weeks after the booster 12/12 (100%) of JE-ADVAX™-vaccinated foals had high serum JEV-neutralizing antibody titres and this neutralizing activity was still present 8.5 months later in 9/12 (75%) foals (Table 1). In contrast, cross-reactivity to MVEV and WNV_KUN_ was generally low or absent (Table 1). Two control foals had sero-converted to flaviviruses at the last sampling, 10 months after trial commencement, presumably due to natural exposure to flaviviruses circulating in South-East Queensland [18,22,23].

### JE-ADVAX™ safety and immunogenicity in pregnant mares

The JE-ADVAX™ vaccine formulation was previously shown to be safe and immunogenic in adult horses [13], however, for licensing purposes it must also be shown to be safe in pregnant animals [21]. Therefore, 17 mares in second trimester of pregnancy were enrolled in a trial, where 11 mares received an initial dose of 12 μg of JEV antigen mixed with 20 mg of ADVAX™ subcutaneously in the neck and four weeks later were boosted with 6 μg of JEV antigen plus ADVAX™. No adverse reactions were noted, other than mild swelling at the inoculation site of some mares 1–3 days following the booster vaccination. Four mares received ADVAX™ only at the two time points and no reactions to the adjuvant were recorded. The remaining two mares received neither antigen nor adjuvant. Pre-vaccination, two of the pregnant mares had flavivirus-specific antibodies, detected in the 4G2-blocking ELISA, however, these antibodies did not neutralize JEV, MVEV or WNV_KUN_ (Table 2). At the time of vaccine boosting (four weeks after initial dose), an additional five mares had developed flavivirus specific antibodies, detected in the blocking ELISA (data not shown), although none had measurable JEV or MVEV neutralizing antibodies. At this time point one of the mares with pre-immunization flavivirus antibodies had developed a WNV_KUN_-neutralizing titre of 80 (Table 2 and data not shown). Four weeks after the booster vaccination, 11/11 (100%) of mares had flavivirus-specific antibodies in the blocking ELISA and of these 8/11 (72%) had developed JEV-neutralizing antibodies, while only four (36%) and three (27%) had MVEV- and WNV_KUN_-neutralizing antibodies, respectively (Table 2). Interestingly, 7.5 months following vaccination all 11 mares (100%) had JEV-neutralizing antibodies, while nine (82%) and two (18%) had neutralizing antibodies to MVEV and WNV_KUN_, respectively (Table 2). This suggests that either some animals responded slowly to the vaccine or had been naturally exposed to flaviviruses, most likely MVEV or Alfuy virus [18,22], thereby providing a boost to the original vaccine response.

The mares foaled 4.5-6 months after the initial vaccination and all had high JEV-neutralizing antibody titres in the colostrum, even though the serum-titres in two of the mares at the time of foaling were below the assay cut-off (Table 3). In 9/11 (82%) of the mares the colostrum antibodies also neutralized MVEV, while in only 2/11 (18%) mares did the colostrum antibodies neutralize WNV_KUN_ (Table 3).

None of the control unvaccinated mares or mares injected with ADVAX™ alone had flavivirus-specific antibodies, based on the pre-vaccination blocking ELISA, however, three of these mares developed flavivirus specific antibodies during the trial period (Table 3). As none of these mares had neutralizing antibodies to JEV, MVEV or WNV_KUN_, it is most likely they sero-converted to some of the other flaviviruses commonly circulating in the area [18]. Interestingly, the colostrum of these three control mares did have neutralizing activity towards JEV and MVEV, but not to WNV_KUN_ (Table 3).

### JE-ADVAX™ responses in foals born to JEV-immune mares

In the second foal trial, 11 foals born to the JE-ADVAX™ vaccinated mares (Tables 2 and 3) received a similar vaccine schedule to that described earlier for the first trial when they were between 36–83 days old. At vaccination, six (55%) foals still had measurable passively transferred maternal serum antibodies to flaviviruses, as detected by the blocking ELISA, although only three (27%) had measurable serum JEV neutralizing titres (Table 4). Nevertheless, all 11/11 (100%) foals responded to the JE-ADVAX vaccine with antibodies detectable by the 4G2-blocking ELISA four weeks after the booster vaccination (Table 4). Of these 10 (91%) had JEV- and 10 (91%) MVEV-neutralizing antibodies after the booster vaccination, but the titres rapidly decreased and by 10 weeks after the booster vaccination only 5/11 (45%) foals had low JEV-neutralizing antibody titres, none had MVEV-specific antibodies and only one had a low WNV_KUN_-specific titre (Table 4). Four foals borne to non-vaccinated mares and left untreated remained flavivirus sero-negative for the duration of the trial (data not shown).

When these 11 vaccinated foals were retested 10 months after the primary vaccination, only two (18%) had flavivirus-specific antibodies detectable in the blocking ELISA, but neither had virus-neutralizing activity (Table 4). The animals, now yearlings, then received a single booster vaccination with 6 μg of JE-ADVAX and were bled two, 5.5, 14 and 20 weeks post booster vaccination. All 11/11 animals were already strongly positive in the flavivirus blocking ELISA two weeks post vaccination, and 11/11 (100%) were confirmed to have JEV-neutralizing antibodies (Table 4), indicating that the original JE-ADVAX vaccination had primed them for a rapid B cell recall response despite interference of maternal antibodies. While the JEV-neutralizing antibody titres declined over the following 18 weeks, 7/11 horses (63%) still had JEV-neutralizing antibody titres > 20 at the last bleed (Table 4). None of the yearlings had MVEV- and WNV_KUN_-neutralizing antibodies at the time of the second booster, but two weeks after the boost 10/11 (91%) horses had MVEV-neutralizing antibodies (Table 4). The yearlings remained negative for neutralizing antibodies to WNV_KUN_ until three months after the booster, when 9/11 (82%) had a detectable titre. The cross-reactive antibody activity to MVEV and WNV_KUN_ had declined below the assay cut-off of < 20 by 20 weeks post booster (Table 4).

## Discussion

The results of this study corroborate and extend those of a previous study by Lobigs et al. [13] and show that the cell culture-derived, inactivated JE-vaccine is safe in horses of any age and when delivered with the novel polysaccharide adjuvant Advax elicits a strong JEV-specific neutralizing antibody response in both pregnant adult horses and in very young foals which lasts at least 10–11 months in the majority of the animals (Tables 1 and 2). While a 3^rd^ booster vaccination was not given at that stage, the results of the vaccine trial in foals with passively acquired immunity suggest that the vaccine delivers robust priming of a memory B-cell response, which results in a strong humoral recall response despite serum antibodies having decreased to undetectable levels (Table 4).

In contrast to the high level of MVEV and WNV cross-protection seen in mice with JE-ADVAX™ vaccine [24] or the live Chimerivax-JE vaccine [25], cross-neutralization of WNV_KUN_ was only present in serum of 27% (3/11) of mares and in 41% (5/12) of foals immunized with two doses of JE-ADVAX™ (Tables 1 and 2). In the foals the low WNV_KUN_ titres detected at day 308 post primary vaccination may even be the result of boosting by natural exposure to JEV sero-complex flaviviruses, as MVEV, WNV_KUN_ and Alfuy virus are known to circulate in the area where the horses were kept [18,22,23]. However, extensive JE-ADVAX dose ranging studies have not yet been performed in horses and hence with a larger vaccine dose higher levels of flavivirus cross-neutralization closer to the levels seen in mice may be achieved [24]. An alternative explanation might be that mice are not ideal models for equine vaccine responses [26].

As could be expected, the JEV-specific antibody titres in colostrum far exceeded those in the serum of the mares at the time of foaling (Table 3). This difference was even more notable for MVEV cross-reactive antibodies, with MVEV-neutralizing activity detectable in the colostrum of 82% (9/11) of immunized mares and in serum of 72% (8/11) of foals 12 h post colostrum uptake, despite being detectable in the serum of only 45% (5/11) of these mares (Tables 3 and 4). While this trial was not designed to assess the half-life of passively acquired immunity, it is nevertheless a notable finding, as it would suggest that vaccination of pregnant mares with the JE-ADVAX vaccine in areas of Australia where MVEV is endemic might confer protection of the foals against this almost invariably fatal infection [22,27-30]. In general, the cross-reactivity to MVEV was greater than that to WNV_KUN_. Nevertheless, the results suggest that it might be possible to further enhance flavivirus cross-protection by additional vaccine boosters, as was seen in the study by Lobigs et al. [13], or increases in vaccine dose.

Two control mares, receiving Advax adjuvant only, were apparently naturally exposed to flavivirus(es) sometime between the start of the trial and foaling, giving rise to high cross-reactivity to JEV and MVEV in their colostrum (Table 3). Similarly, a control mare that received neither vaccine nor adjuvant, had high JEV- and MVEV-neutralizing antibody titres in its colostrum despite only having serum neutralizing activity to Kokobera virus (titre of 40 [18]), which normally does not confer cross-protection to JEV serocomplex viruses [18,31]. Two of the foals born to these three control mares were subsequently positive for flavivirus antibodies in the 4G2-blocking ELISA for a couple of months, but at no point was JEV- or MVEV-neutralizing activity detected in their serum (data not shown). This aspect was not further pursued, but it might be speculated that these were antibodies of low affinity and/or avidity and/or quickly catabolized in the foals.

The degree of interference with immune responses in young animals by passively acquired maternal antibodies may depend on the animal species, antigen type, route of vaccination and other variables [32-38]. While all the foals received flavivirus-specific antibodies in colostrum, resulting in detectable serum JEV-neutralizing antibodies in 91% of foals at 12 h post suckle, this neutralizing activity decreased to below detection level in all but the three youngest foals by the time of primary vaccination (Table 4), and all but one foal responded with moderately high to high JEV-neutralizing titres four weeks after the second vaccine dose. However, in the presence of maternal antibodies the antibody response to vaccination was shorter-lived, with only 5/11 (45%) foals still having JEV-neutralizing antibodies 10 weeks after the booster. Six months later all but two were negative for flavivirus antibodies in the 4G2-blocking ELISA, but all 11 animals, by then yearlings, responded to a second booster with a very rapid and robust antibody response. Thus, while vaccination at an age where passively acquired antibodies were still present prevented sustained serum neutralizing antibody responses, memory B cells were still induced in these foals immunized in the presence of passive antibodies, as reflected in their vigorous response to a single vaccine boost. This suggests that the apparent “window of susceptibility” created by vaccination in the presence of passively acquired antibodies, may not in reality be as much of a problem as generally thought [35,37-39], as even in the absence of pre-existing serum antibody, the primed memory B cell response is able to respond rapidly enough to control any infection. Arthropod-borne viruses initially replicate at the site of inoculation before spreading haematogenously or via the lymphatic system to local lymph nodes and beyond [40,41]. While the route and mechanisms of neuro-invasion by the encephalitic flaviviruses are still unknown [42], we recently described a case of MVEV-encephalitis in a horse, in which MVEV-neutralizing antibodies were present one week prior to clinical symptoms, but not two weeks prior to clinical disease [22]. This suggests that even in a primary infection the antibody response to JEV-serocomplex viruses may be relatively fast, and can be anticipated to be even faster in a recall response [43]. If this assumption is correct, then primed animals lacking detectable serum-neutralizing antibodies might still be protected by a rapid memory B cell recall response able to neutralize the virus before it spreads to the central nervous system. This is similar to the results obtained in JE-ADVAX-immunized beta-2-microglobulin knockout mice, which were still protected against JEV, despite having no detectable serum neutralizing antibody pre-challenge, thanks to a robust recall response and rapid rise in serum antibody titer in response to the challenge virus [44]. Future studies should aim to test this hypothesis in horses, since as discussed above, the murine immune response may not truly reflect that of equines [26], nor does the disease progression in mice reflect that seen in natural and experimental infections of horses with JEV, MVEV and WNV_KUN_ ([22,45-49]; Bielefeldt-Ohmann et al., unpublished data 2013).

In conclusion, JE-ADVAX™ was safe, well-tolerated and highly immunogenic in both young foals and in pregnant mares, in which it induced high titre JEV-specific antibodies in colostrum, thus ensuring passive transfer of protective antibodies to the newborn foals. Despite evidence of maternal antibody interference, foals of immune dams developed strong memory B cell responses to JEV, as reflected in a robust recall response to a single booster JE-ADVAX dose as yearlings. Although primarily designed to provide protection against JEV, some cross-neutralisation against MVEV and WNV was seen in some of the JE-ADVAX immunized horses. Future studies will test modifications to the vaccination protocol, including increasing the antigen or adjuvant dose, adding a further booster immunization, priming with JE-ADVAX and boosting with already licensed WNV vaccines [47,50] or with novel WNV vaccine candidates [48,51-54] to see whether it is possible to induce cross-protection against a wider spectrum of flaviviruses.
